# Monitoring and Outcomes of Central Line-Associated Bloodstream Infections in a Tertiary Care Intensive Care Unit

**DOI:** 10.7759/cureus.63428

**Published:** 2024-06-28

**Authors:** Peter B Kharduit, Kaustuv Dutta, Clarissa J Lyngdoh, Prithwis Bhattacharyya, Valarie Lyngdoh, Annie B Khyriem, Suriya K Devi

**Affiliations:** 1 Microbiology, North Eastern Indira Gandhi Regional Institute of Health and Medical Sciences, Shillong, IND; 2 Anesthesiology, North Eastern Indira Gandhi Regional Institute of Health and Medical Sciences, Shillong, IND; 3 Anesthesiology and Critical Care, North Eastern Indira Gandhi Regional Institute of Health and Medical Sciences, Shillong, IND; 4 Clinical Microbiology, North Eastern Indira Gandhi Regional Institute of Health and Medical Sciences, Shillong, IND

**Keywords:** multidrug-resistant organisms, device utilization ratio, infection control, surveillance, central line-associated bloodstream infection

## Abstract

Background

Central line-associated bloodstream infections (CLABSIs) are significant healthcare-associated infections that increase morbidity, mortality, and healthcare costs. This study aims to analyze the frequency, microbiology, risk factors, and outcomes of CLABSI in an adult intensive care unit.

Methods

We conducted a hospital-based, prospective surveillance study in the critical care unit of a tertiary care hospital. We included patients with a central line (CL) from admission until discharge or line removal. Data collection focused on patient demographics, comorbidities, CL insertion site, and CLABSI rates. The incidence of CLABSI was calculated per 1,000 CL-days, and statistical analysis was performed using the Chi-square test.

Results

Of the 169 patients enrolled, 123 episodes of bloodstream infections were recorded, 56 (45.5%) of which were CLABSIs. The organisms most frequently isolated were *Klebsiella pneumoniae* (n = 14; 24.6%), *Enterobacter cloacae complex* (n = 11; 19.3%), *Klebsiella* species (n = 7; 12.28%), and *Acinetobacter baumannii* (n = 7; 12.28%). The overall CLABSI rate was 24.70 per 1,000 CL-days. No significant association was found between CLABSI and patient age, gender, or the site of CL insertion. However, a significant relationship was observed between CLABSI and the presence of comorbid conditions (p = 0.001). The study also noted a high rate of antibiotic resistance among the isolated pathogens.

Conclusions

Our results emphasize the need for stringent infection control measures and suggest that comorbid conditions significantly increase the risk of CLABSI. Addressing antibiotic resistance and implementing effective prevention strategies are essential for reducing the burden of CLABSIs.

## Introduction

A central venous catheter (CVC), or a central line (CL), is an intravascular catheter with the tip positioned in the proximal third of the superior vena cava, right atrium, or inferior vena cava. Approximately five million CVCs are inserted annually to administer medications, intravenous fluids, parenteral nutrition, and other treatments [1,2]. CLs can experience various complications, such as malfunction and infection [3]. Infections that enter the bloodstream can lead to bacteremia and septicemia, especially in hospitalized and debilitated patients [4].

The United States National Healthcare Safety Network (NHSN) defines a CL-associated bloodstream infection (CLABSI) as a laboratory-confirmed bloodstream infection (LCBI) in which a microorganism common to CLs is identified, with the CL in place at the LCBI date of event or the day before [5]. If blood cultures are positive for normal skin flora, CLABSI identification requires at least two pairs of positive blood cultures and one or more clinical symptoms, such as fever, chills, or hypotension. CLABSIs lead to prolonged hospitalization, increased healthcare costs, and mortality. Annually, approximately 250,000 cases of bloodstream infections (BSIs) occur in the United States, most of which are associated with the use of CLs [6].

The incidence rate of CLABSI is calculated as the number of CLABSI cases per 1,000 CL-days [5]. The rate in the United States is estimated to be 0.8 [7]. The mortality rate from CLABSIs is approximately 15% [8]. In India, the incidence of CLABSI varies considerably in different studies. Mehta et al. reported a CLABSI rate of 7.9 per 1000 catheter days in 12 ICUs of seven hospitals, whereas in a study by Mathur P et al. involving a network of 27 tertiary care hospitals, 45.7% were classified as CLABSIs [9,10].

Several risk factors are associated with infections related to CL use. The site of insertion and a prolonged catheter dwell time are significant factors contributing to infectious complications [11,12]. Tunneled catheters are associated with fewer CLABSI cases than non-tunneled catheters. Peripherally inserted central catheters (PICCs) are associated with a lower risk of CLABSI, likely due to the lower density of bacterial colonization at the mid-arm insertion site [13]. Multi-lumen CVCs, while convenient for various patient care purposes, may provide additional routes for infection [14].

CLABSI is preventable, and reducing CLABSI events can be achieved by implementing prevention strategies with significant compliance [15]. This study aimed to determine the frequency, microbiology, risk factors, and outcomes of CLABSI in an adult ICU at a tertiary care center.

## Materials and methods

This study was a hospital-based, prospective surveillance study conducted in the critical care unit of North Eastern Indira Gandhi Regional Institute of Health and Medical Sciences (NEIGRIHMS), a tertiary care institute in Shillong, Meghalaya, in the northeastern part of India, from June 2021 to May 2022. We obtained informed consent from each patient. The study included patients with a CL from admission until the day of discharge or CL removal, whichever occurred first. We collected patient details such as age, gender, presence of comorbidities, and the site of insertion to determine site-specific infection rates; we also noted patient outcomes, the organisms isolated, and their antimicrobial susceptibility patterns. Patients with other devices, such as arterial catheters, arteriovenous fistulas, arterio-venous grafts, and atrial catheters, were excluded from the study.

We monitored the rates of CLABSIs using surveillance methods consistent with the Centers for Disease Control and Prevention/National Healthcare Safety Network (NHSN) guidelines [5]. An eligible BSI organism meets the criteria for an LCBI.

The criteria for identifying LCBIs are defined in two categories. LCBI 1 pertains to patients of any age with a recognized bacterial or fungal pathogen, which is not included on the NHSN commensal list. This pathogen must be identified from one or more blood specimens obtained by culture and must not be related to an infection at another site. LCBI 2 involves patients of any age with an NHSN common commensal identified by culture from two or more blood specimens. These patients must exhibit at least one of the following signs or symptoms: fever greater than 38.0 °C, chills, hypotension, hypothermia below 36.0°C, apnea, or bradycardia. Furthermore, the organism isolated in the blood sample must not be related to an infection at another site. The matching blood cultures for LCBI 2 must either be collected on separate occasions, with the second sample taken on the same day or the next day, collected simultaneously but from different sites, or collected from the same site through two separate blood draws, each utilizing a separate needle and syringe.

We calculated the incidence of CLABSI as the CLABSI rate per 1,000 CL-days. The device utilization ratio (DUR) was determined using the following formula: number of device days divided by the number of patient days.

Ethics

The study was carried out following ethical clearance from the Institutional Ethics Committee (IEC), project number T81/2021/81. The procedures followed were in accordance with the ethical standards of the responsible committee on human experimentation (institutional or regional) and with the Helsinki Declaration of 1975, as revised in 2000.

Statistical analysis

We analyzed the patient information data using the Chi-square test with IBM SPSS Statistics for Windows, version 29.0 (released 2023, IBM Corp., Armonk, NY). We expressed the causative agents of CLABSI as simple percentages. In addition, we recorded the outcomes for patients with and without CLABSI and reported the crude mortality rate as a simple percentage.

## Results

During the surveillance period, we enrolled 169 patients with CLs in the study. We identified 123 episodes of BSIs, which included 56 episodes of CLABSIs in 52 patients, accounting for 45.5% of the total BSIs. In addition, there were 54 primary BSIs (43.9%) and 13 episodes of secondary BSIs (10.6%). The overall BSI rate during this study period was 14.6 per patient-days, and the CLABSI rate was 24.70 per 1,000 CL-days (Table 1).

**Table 1:** Incidence of BSI Abbreviation: BSI, bloodstream infection; CL, central line; CLABSI, central line-associated bloodstream infection

Time period	Patient-days	CL-days	CLABSI (N)	Non-CLABSI (N)	Secondary (N)	Total BSI Rate	CLABSI rate
Jun-21	523	131	10	6	0	30.59	76.34
Jul-21	569	77	2	9	3	24.6	25.97
Aug-21	626	161	5	6	0	17.57	31.06
Sep-21	701	263	7	3	2	17.12	26.62
Oct-21	667	120	7	8	1	23.99	58.33
Nov-21	725	145	4	6	0	13.79	27.59
Dec-21	796	186	4	4	2	12.56	21.51
Jan-22	782	247	4	2	1	8.95	16.19
Feb-22	719	249	2	1	0	4.17	8.03
Mar-22	816	205	2	2	3	8.58	9.76
Apr-22	776	226	4	5	1	12.89	17.7
May-22	739	257	5	2	0	9.47	19.46
Total	8439	2267	56	54	13		

Of the 56 episodes of CLABSI, 57 organisms were isolated. The most common organism was *Klebsiella pneumoniae* (n = 14; 24.6%), followed by* Enterobacter cloacae* complex (n = 11; 19.3%), *Klebsiella* species (n = 7; 12.28%) and *Acinetobacter baumannii *(n = 7; 12.28%; Table 2).

**Table 2:** Distribution of organisms isolated from CLABSI cases Abbreviation: CLABSI, central line-associated bloodstream infection

Organism	Frequency	Percentage
Klebsiella pneumoniae	14	24.56
*Enterobacter cloacae *complex	11	19.3
Klebsiella species	7	12.28
Acinetobacter baumannii	7	12.28
Pseudomonas aeruginosa	4	7.02
Staphylococcus aureus	4	7.02
Klebsiella aerogenes	3	5.26
Acinetobacter lwoffii	3	5.26
Escherichia coli	2	3.51
Pseudomonas fluorescens	1	1.75
Pseudomonas putida	1	1.75
Total	57	100.0

The mean age of patients with CLABSI was 51.8 (standard deviation (SD) = 17.8), while the mean age of patients without CLABSI was 48.4 (SD = 16.3). The association of CLABSI with age in this study was not significant (p = 0.276). The distribution of CLABSI by gender showed 22 women (42.3%) and 30 men (57.7%), while among the 117 catheterized patients without BSI or with non-CLABSI BSI, there were 52 women (44.4%) and 65 men (55.6%). There was no significant gender association with CLABSI in this study (p = 0.718).

Regarding the site of CL insertion, the most common was jugular for both CLABSI cases (n = 28; 50%) and non-CLABSI cases (n = 73; 64.6%). The overall distribution of CL sites was 101 jugular (59.8%), 40 subclavian (23.4%), and 28 femoral (16.6%). There was no significant association between the occurrence of CLABSI and the site of insertion (p = 0.184; Tables 3, 4).

**Table 3:** Insertion site distribution among CLABSI and non-CLABSI cases Abbreviation: CLABSI, central line-associated bloodstream infection

Insertion site		CLABSI incidence		Total	P-value
		YES	NO		
Femoral	Frequency	12	16	28	0.184
	Percentage	21.4%	14.2%	16.6%	
Subclavian	Frequency	16	24	40	
	Percentage	28.6%	21.2%	23.7%	
Jugular	Frequency	28	73	101	
	Percentage	50%	64.1%	59.8%	
Total	Frequency	56	113	169	
	Percentage	33.1%	66.9%	100.0%	

**Table 4:** Statistical analyses of insertion site distribution among CLABSI and non-CLABSI cases Abbreviation: CLABSI, central line-associated bloodstream infection

Analysis	Value	Df	Asymp. sig. (two-sided)
Pearson chi-square	3.381	2	0.184
Likelihood ratio	3.349	2	0.187
Linear-by-linear association	0.000	1	0.995
Number of valid cases	169		

A total of 44 patients (26%) in this study had associated comorbid conditions, such as hypertension, diabetes, chronic kidney disease, and others. CLABSI occurred in 22 patients with comorbidities (42.3%) (Table 5). There was a significant association of CLABSI with comorbid conditions (p = 0.001; Tables 5, 6).

**Table 5:** Distribution of comorbid conditions in CLABSI cases Abbreviation: CLABSI, central line-associated bloodstream infection

Comorbid conditions	Incidence of CLABSI		Total	P-value
	YES	NO		
Yes, n (%)	22 (42.3%)	22 (18.8%)	44 (26%)	0.001
No, n (%)	30 (57.7%)	95 (81.2%)	125 (74%)	
Total, n (%)	52 (30.8%)	117 (69.2%)	169 (100%)	

**Table 6:** Statistical analyses of comorbid conditions with CLABSI a. 0 cells (0.0%) have an expected count of less than 5. The minimum expected count is 13.54. b. Computed only for a 2 x 2 table. Abbreviation: CLABSI, central line-associated bloodstream infection

Analysis	Value	Df	Asymp. sig. (two-sided)	Exact. sig. (two-sided)	Exact. sig. (one-sided)
Pearson chi-square	10.328^a^	1	0.001		
Continuity correction^b^	9.143	1	0.002		
Likelihood ratio	9.861	1	0.002		
Fisher's exact test				0.002	0.002
Linear-by-linear association	10.267	1	0.001		
Number of valid cases	169				

The mean duration of CL indwelling among the CLABSI cases was 10.00 days (SD = 8.985), whereas the mean duration in the BSI (non-CLABSI) and non-BSI cases was 7.29 days (SD = 6.100; Table 7).

**Table 7:** Central line indwelling days in CLABSI Abbreviation: CLABSI, central line-associated bloodstream infection

Incidence of CLABSI	Duration of central line in-dwelling		Standard deviation
	Days, n	Mean days	
YES	56	10.00	8.985
NO	113	7.287	6.100

Twelve (7.1%) of the 169 patients with CLs had their lines changed at least once during their admission to the ICU. Among the patients with CLABSIs, nine (16.1%) had their CLs changed at least once. By contrast, only three (2.7%) of 113 patients with non-BSI or BSI (non-CLABSI) cases had their lines changed. There was a significant association between the change of CLs and the occurrence of CLABSI (p = 0.001; Table 8).

**Table 8:** Change of central line, CLABSI, and other catheter patients Abbreviation: CLABSI, central line-associated bloodstream infection

Change of central line		Incidence of CLABSI		Total	P-value
		Yes	No		
YES	Frequency	9	3	12	0.001
	Percentage	16.1%	2.7%	7.1%	
NO	Frequency	47	110	157	
	Percentage	83.9%	97.3%	92.9%	
Total	Frequency	56	113	169	
	Percentage	33.1%	66.9%	100.0%	

Mortality among the patients with CLABSIs was observed in 18 (34.7%) of 52 patients, whereas among 117 non-BSI and BSI (non-CLABSI) patients, 51 (43.6%) died in the ICU (p = 0.273; Tables 9, 10). The DUR during the study period was 0.27 (Table 11).

**Table 9:** Mortality cases among CLABSI and non-CLABSI cases Abbreviation: CLABSI, central line-associated bloodstream infection

Incidence of CLABSIs		Occurrence of death		Total	P-value
		Yes	No		
YES	Frequency	18	51	69	0.273
	Percentage	34.7%	43.6%	40.8%	
NO	Frequency	34	66	100	
	Percentage	65.3%	56.4%	59.2%	
Total	Frequency	52	117	169	
	Percentage	30.8%	69.2%	100.0%	

**Table 10:** Statistical analyses of mortality outcomes associated with CLABSI *0 cells (0.0%) have an expected count of less than 5. The minimum expected count is 20.22 Abbreviation: CLABSI, central line-associated bloodstream infection

	Value	Df	Asymp. sig. (two-sided)	Exact. sig. (two-sided)	Exact. sig. (one-sided)
Pearson chi-square	1.200*	1	0.273		
Continuity correction	.857	1	0.354		
Likelihood ratio	1.214	1	0.271		
Fisher's exact test				0.311	0.177
Linear-by-linear association	1.193	1	0.275		
Number of valid cases	169				

**Table 11:** CLABSI rate and DUR Abbreviations: CLABSI, central line-associated bloodstream infection; DUR, device utilization ratio

Month-year	Patient-days	Central line-days	CLABSI rate	DUR
Jun-21	523	131	76.34	0.25
Jul-21	569	77	25.97	0.14
Aug-21	626	161	31.06	0.26
Sep-21	701	263	26.62	0.38
Oct-21	667	120	58.33	0.18
Nov-21	725	145	27.59	0.2
Dec-21	796	186	21.51	0.23
Jan-22	782	247	16.19	0.32
Feb-22	719	249	8.03	0.35
Mar-22	816	205	9.76	0.25
Apr-22	776	226	17.7	0.29
May-22	739	257	19.46	0.35
Total	8439	2267		

The antimicrobial sensitivity test of the isolates was performed as per Kirby-Bauer disk diffusion method and testing for colistin sensitivity was done using the broth microdilution method. Of the four *Staphylococcus aureus *isolates, two (50%) were methicillin-resistant *Staphylococcus aureus *(MRSA). All four (100%) isolates showed sensitivity to gentamicin, chloramphenicol, and vancomycin, while they were resistant to penicillin, ofloxacin, erythromycin, and clindamycin, and two (50%) isolates showed resistance to cotrimoxazole (Table 12).

**Table 12:** Antimicrobial sensitivity pattern of Staphylococcus aureus Abbreviations: S, sensitive; R, resistant; I, intermediate

Antibiotic	S	Percentage	R	Percentage	I	Percentage
Ciprofloxacin	2	50%	1	25%	1	25%
Cefoxitin	2	50%	2	50%	0	0.0%
Clindamycin	0	0.0 %	4	100.0%	0	0.0%
Cotrimoxazole	2	50%	2	50%	0	0.0%
Chloramphenicol	4	100%	0	0.0%	0	0.0%
Erythromycin	0	0.0 %	4	100.0%	0	0.0%
Gentamicin	4	100.0%	0	0.0%	0	0.0%
Ofloxacin	0	0.0%	4	100.0%	0	0.0%
Penicillin	0	0.0%	4	100.0%	0	0.0%
Vancomycin	4	100.0%	0	0.0%	0	0.0%

All 14 isolates of *Klebsiella pneumoniae* were resistant to amoxicillin-clavulanate. Ten of the isolates (71.4%) were sensitive to piperacillin-tazobactam, meropenem, imipenem, and ertapenem. All seven isolates of *Klebsiella* species and the three isolates of *Klebsiella aerogenes *were resistant to amoxicillin-clavulanate, tetracycline, cefuroxime, ampicillin-sulbactam, cefixime, and cefazolin. All the isolates of *Klebsiella aerogenes *and five (71.4%) isolates of *Klebsiella* species were also resistant to piperacillin, ciprofloxacin, ofloxacin, cefotaxime, ceftriaxone, ceftazidime, and chloramphenicol. All *Enterobacter cloacae* complex isolated were resistant to ciprofloxacin, tetracycline, ceftazidime, and cefixime, and nine (81.8%) were sensitive to amikacin and gentamicin. Only three (27.3%) isolates were sensitive to cefotaxime, ceftriaxone, and cefoperazone. Six (71.4%) of *Acinetobacter baumannii *isolates were sensitive to ofloxacin, amikacin, cotrimoxazole, piperacillin-tazobactam, and minocycline, with four (57.1%) being sensitive to imipenem and meropenem. Two of the seven *Acinetobacter baumannii *isolates (28.6%) were multidrug-resistant. All four isolates of *Pseudomonas aeruginosa *were sensitive to ciprofloxacin, ofloxacin, and cefepime, and three (75%) were sensitive to piperacillin, ceftazidime, meropenem, imipenem, and piperacillin-tazobactam, with two (50%) being sensitive to amikacin and gentamicin.

## Discussion

CLABSIs are preventable healthcare-associated infections that lead to increased healthcare costs, morbidity, and mortality. CLABSIs account for approximately 28,000 deaths annually in the United States alone and impose a financial burden amounting to billions of US dollars each year [16].

Over a 12-month study period, we recorded 123 episodes of BSI, among which 56 episodes (45.5%) were CLABSIs from 52 CL patients, 54 episodes were primary BSIs (43.9%), and 13 were secondary BSIs (10.6%; Tables 1, 2). This distribution is similar to findings by Mathur et al., who reported that 45.7% of BSIs were CLABSIs, 35.9% were primary BSIs not associated with a CL, and 18.5% were secondary BSIs [10]. Our overall BSI rate was 14.6 per 1000 patient-days, and the CLABSI rate was 24.70 per 1,000 CL-days, calculated over 8,439 patient-days and 2,267 CL-days. This incidence is notably higher than other studies, such as one conducted by Maqbool S et al. in a medicine ICU, which reported a CLABSI incidence of 9.3 per 1,000 CL-days [12,17].

The peak CLABSI rate during June 2021 was 76.34 per 1,000 LC-days, significantly influenced by patients' post-coronavirus disease 2019 (COVID-19) complications. This was attributed to the lack of trained manpower, increased number of patients, shortages of personal protective equipment, and reduction in the activities for maintenance of the CVCs, like scrub-the-hub compliance. Non-adherence to the timely removal of the CL was another factor. Trained ICU staff were also shifted from the ICU surveillance unit to other COVID areas resulting in a lack of supervision in the surveillance unit. Some expressed that they were too busy to perform hand hygiene. This surge correlated with increased CLABSI rates reported by Ben-Aderet et al., who found a CLABSI rate of 6.31 per 1,000 CL-days among COVID-19 ICU patients, compared to 0.60 in non-COVID-19 patients, indicating a relative risk of 10.5 for CLABSI [18]. This underlines the importance of training, provision of resources, and enhancement of motivation to improve infection control activities. 

In this study, 57 organisms were isolated from 56 CLABSI episodes, predominantly Gram-negative organisms like *Klebsiella pneumoniae* (24.56%), followed by *Enterobacter cloacae *complex, *Klebsiella species*, and *Acinetobacter baumannii*. Mathur et al. reported similar findings with *Klebsiella* spp. as the most common pathogen [10].

The mean age of patients with CLABSIs was 51.8, and no significant association was found between age and CLABSI development (p = 0.276). Lafuente Cabrero et al. also reported no significant association between age and CLABSI [19]. Gender distribution in CLABSI cases was also not significant in our study (p = 0.718), echoing findings by Alotaibi et al., at King Saud University Medical City in Riyadh, where no relationship was found between gender and CLABSI [20]. This finding can prevent any potential gender or age bias in CLABSI surveillance.

In our 56 CLABSI cases, the CLs were inserted in the jugular (101; 50%), subclavian (40; 28.6%), and femoral (28; 21.4%) sites, with no significant association found between the site of insertion and CLABSI occurrence (p = 0.184). Similarly, Marik et al., in their randomized controlled trials, found no significant risks associated with the site of CL insertion [21]. The authors opined that although femoral vein access is to be avoided and it is also recommended as a part of the CL insertion bundle, its impact on the occurrence of CLABSI remains unclear [21-23]. In our study, thorough insertion and maintenance care was given to the femoral sites, which probably resulted in the lack of association between the site of insertion and the occurrence of CLABSI.

Comorbid conditions like diabetes, hypertension, and chronic kidney diseases were present in 22 (42.3%) of the patients who developed CLABSIs. This study found a significant association between the presence of comorbid conditions and CLABSI (p = 0.001). Similar findings were seen in other studies [17,20]. 

The mean duration of CL indwelling among the CLABSI cases was 10.00 days (SD = 8.985), compared to 7.287 days (SD = 6.100) in 113 BSI (non-CLABSI) and non-BSI cases. In our study, we found that prompt removal of catheters was not being followed for all the cases. The CVC lines were not removed since they were convenient for blood draws and infusions. Efforts should be made toward the daily assessment of CL through checklists and encouraging the prompt removal of unnecessary lines. Pitiriga et al., in a retrospective study, showed that an increased duration of central venous access was associated with a gradually higher rate of CLABSI events [24].

Of the 169 patients included in this study, 69 (40.8%) had fatal outcomes. Among the patients with CLABSIs, mortality was observed in 18 (34.7%) of 52, whereas of the 117 BSI (non-CLABSI) and non-BSI patients, 51 (43.6%) died in the ICU (Tables 9, 10). However, there was no significant association between CLABSI and fatal outcomes (p = 0.273). Alshahrani et al., in a systematic study within the past 13 years, found that the overall mortality rate was 11.1%, with CLABSI cases having a mortality rate of 12.9% and non-CLABSI cases having a mortality rate of 10.7%. [25]. CLABSI is associated with a significantly increased risk of death, yet in our study, more deaths have occurred in the non-CLABSI cases. Other risk factors should be investigated including the patient sub-groups and comorbidities. 

Of the four isolates of *Staphylococcus aureus* in this study, two (50%) were MRSA. Singhal et al. reported a 37.0% prevalence of MRSA among 752 isolates in a prospective observational study conducted across adult, pediatric, and neonatal ICUs from 2011 to 2018 [26]. Among the 14 isolates of *Klebsiella pneumoniae*, all showed resistance to amoxicillin-clavulanate, but 10 (71.4%) were sensitive to piperacillin-tazobactam and carbapenems. Only four (28.6%) of these isolates were sensitive to other antibiotics, including ciprofloxacin and gentamicin. Inamdar et al. reported that all isolates of *Klebsiella pneumoniae* showed resistance to ciprofloxacin, amoxicillin-clavulanic acid, and third-generation cephalosporins [27]. Agrawal et al., in a retrospective study at the medical/surgical oncology department, found that most Gram-negative isolates were extended-spectrum beta-lactamase producers [28].

All four isolates of *Pseudomonas aeruginosa* were sensitive to ciprofloxacin, ofloxacin, and cefepime, and three (75%) were sensitive to piperacillin, ceftazidime, meropenem, imipenem, and piperacillin-tazobactam. Tabaseera et al. reported that only one of nine bacterial isolates of CLABSIs was susceptible to all tested antibiotics, including amikacin and carbapenems [29]. Among the seven isolates of *Acinetobacter baumannii*, six (71.4%)were sensitive to ofloxacin, amikacin, cotrimoxazole, piperacillin-tazobactam, and minocycline; four (57.1%) were sensitive to imipenem and meropenem, and two of the seven isolates (28.6%) were multidrug-resistant organisms (MDROs). They were resistant to at least one antimicrobial agent in three or more antimicrobial categories. Alwazzeh et al., in a study showing a six-year trend of CLABSI pathogens, found that there is a predominance of Gram-negative pathogens that are highly multidrug-resistant [30].

This study has several limitations that should be considered when interpreting the results. First, it was conducted at a single tertiary care center, which may limit the generalizability of the findings to other settings or populations. Second, the exclusion of patients with other devices such as arterial catheters, arteriovenous fistulas, and atrial catheters might have influenced the overall infection rates and the comprehensiveness of our analysis. The study also did not account for the potential impact of varying infection control practices or interventions that might have been implemented during the study period. Lastly, the lack of long-term follow-up data precludes an assessment of the extended outcomes and complications related to CLABSI. Despite these limitations, the study provides valuable insights into the epidemiology, microbiology, and risk factors associated with CLABSI in a critical care setting. Future multi-center studies with broader inclusion criteria and standardized infection control protocols are recommended to validate and extend these findings.

## Conclusions

Despite CLs serving various critical purposes in the management of critically ill patients, they are associated with numerous complications, including infectious ones. CLABSIs remain a significant healthcare-associated infection, leading to severe morbidity, prolonged hospital stays, substantial financial burden, and potentially fatal outcomes. The rate of CLABSIs in this study was notably high, particularly during certain months. Early implementation of robust infection prevention and control measures, with regular assessments to ensure strict adherence, is imperative. This study also highlights the presence of MDROs, which may guide future patient management and necessitate immediate infection control practices.
